# Inferring latent task structure for Multitask Learning by Multiple Kernel Learning

**DOI:** 10.1186/1471-2105-11-S8-S5

**Published:** 2010-10-26

**Authors:** Christian Widmer, Nora C Toussaint, Yasemin Altun, Gunnar Rätsch

**Affiliations:** 1 Friedrich Miescher Laboratory, Max Planck Society, Spemannstr. 39, 72076 Tübingen, Germany; 2 Center for Bioinformatics Tübingen, Eberhard-Karls-Universitüt, Sand 14, 72076 Tübingen, Germany; 3 Max Planck Institute for Biological Cybernetics, Spemannstr. 38, 72076 Tübingen, Germany

## Abstract

**Background:**

The lack of sufficient training data is the limiting factor for many Machine Learning applications in Computational Biology. If data is available for several different but related problem domains, Multitask Learning algorithms can be used to learn a model based on all available information. In Bioinformatics, many problems can be cast into the Multitask Learning scenario by incorporating data from several organisms. However, combining information from several tasks requires careful consideration of the degree of similarity between tasks. Our proposed method simultaneously learns or refines the similarity between tasks along with the Multitask Learning classifier. This is done by formulating the Multitask Learning problem as Multiple Kernel Learning, using the recently published *q*-Norm MKL algorithm.

**Results:**

We demonstrate the performance of our method on two problems from Computational Biology. First, we show that our method is able to improve performance on a splice site dataset with given hierarchical task structure by refining the task relationships. Second, we consider an MHC-I dataset, for which we assume no knowledge about the degree of task relatedness. Here, we are able to learn the task similarities* ab initio* along with the Multitask classifiers. In both cases, we outperform baseline methods that we compare against.

**Conclusions:**

We present a novel approach to Multitask Learning that is capable of learning task similarity along with the classifiers. The framework is very general as it allows to incorporate prior knowledge about tasks relationships if available, but is also able to identify task similarities in absence of such prior information. Both variants show promising results in applications from Computational Biology.

## Background

In Machine Learning, model quality is most often limited by the lack of sufficient training data. In presence of data from different but related tasks, it is possible to boost the performance of each task by leveraging all available information. Multi-task learning (MTL), a subfield of Machine Learning, considers the problem of inferring models for each task simultaneously while imposing some regularity criteria or shared representation in order to allow learning across tasks. There has been an active line of research exploring various methods (e.g. [1,2]), providing empirical findings [3] and theoretical foundations [4,5]. Most of these methods assume uniform relations across tasks. However, it is conceivable to leverage MTL methods by taking into account the degree of relatedness among tasks. Recently, this direction has been investigated in the context of hierarchies [6,7] and clusters [8] of tasks, where the relation across tasks as well as the models for each task are inferred simultaneously.

In this paper, we follow this line of research and investigate Multitask Learning scenarios where there exists a latent structural relation across tasks. In particular, we model the relatedness between tasks by defining *meta*-tasks. Here, each meta-task corresponds to a subset of all tasks, representing the common properties of the tasks within this subset. Then, the model of each task can be derived by a convex combination of the meta-tasks it belongs to. Moreover, the latent structure over tasks can be represented by a collection of the meta-tasks. Information is transferred between two tasks *t*, *t′* with respect to their relatedness according to the latent structure (number of meta-tasks in which *t*,* t′* co-occur and the importance of each of these meta-tasks defined by the mixture weights).

Clearly, such an approach is highly sensitive to the chosen structure and in the absence of prior knowledge, learning the latent structure is a crucial component of MTL with non-uniform relatedness. Starting from a special case, where there exists a single meta-task consisting of all tasks (standard MTL), we show that inferring the latent structure can be cast as a Multiple Kernel Learning problem, where the base kernels are defined with respected to* Dirac* kernels [9] that establish relatedness of all possible task combinations and hence correspond to all possible meta-tasks.

Kernel methods are used in a wide-range of problems, as the kernel abstracts the input space from the Machine Learning algorithm. One can use several kernels to incorporate different aspects of the same instance (e.g. genomic sequence data and data from blood measurements for one patient) and combine them into the same optimization problem. Multiple Kernel Learning can be used to determine the combination of kernels that is best for the problem at hand. This is done by learning an optimal weighting of the individual kernels along with training a predictor.

Our contribution is the combination of MTL and MKL to address the central question in Multitask Learning, of how to identify the relationships between tasks and to translate them into meaningful parameters in the formulation of the used learning algorithm. We show that MKL can be used to 1) refine a given hierarchical structure that relates the tasks at hand and 2) to identify subsets of tasks for which information transfer pays off in absence of prior information on task relations.

Besides applications in Natural Language Processing [10] and Medical Domains, Multitask Learning is particularly relevant to Computational Biology. In this setting, tasks often correspond to organisms, giving rise to a whole range of problems. The fact that the availability of data describing the same biological mechanism in several organisms is a reoccurring theme makes the Multitask Learning approach particularly well suited for many applications in the field. There has been previous work using Domain Adaptation (closely related to Multitask Learning) in the context of splice site prediction [3]. Furthermore, it was shown [9] that Multitask Learning can be used to leverage the state-of-the-art in peptide MHC-I binding prediction, which is a problem relevant for vaccine design. Given the success of MTL in Computational Biology and highly structured relation across organisms (tasks), we apply our method to two important Computational Biology problems, namely MHC-I binding prediction and splice site prediction. The competitiveness of our results shows the validity of our approach.

## Preliminaries

In a single-task supervised learning scenario, a sample of example-label pairs *D* = {(*x_i_,y_i_*)}*_i=_*_1,…,_*_n_* is given, where the *x_i_* live in an input space *X* and *y_i_* ∊ {−1,1} (for binary classification). The goal is to learn a function *f* such that *f*(*x_i_*) ≈ *y_i_* that generalizes well to unseen data.

Before we describe our formulation of MTL as MKL approach, we briefly review the formulations of MTL and MKL that lay the foundations for our approach.

### Multitask Learning

In MTL [1], we are given one labeled sample *D_t_* for each of *T* tasks. Similar to the single-task supervised learning scenario, we are now interested in obtaining *T* hypotheses *f_t_*, one for each task.

We will formulate our method based on the Support Vector Machine (SVM), which has proven to generalize well [11], scales to large amounts of training data [12,13] and is able to incorporate arbitrary data sources by means of kernels (e.g., [14]). The generalization to other learning approaches appears straight-forward as we mainly consider the extension of kernels to reflect task similarity, although details regarding the learning of their linear combination may differ.

Therefore, we start out with a regularization-based Multitask Learning method that was similarly proposed in the context of SVMs [2,10,15]. The basic idea is that models **w***_t_* are learned simultaneously for all tasks. Information transfer between tasks is achieved by sharing a general component  and enforcing similarity of each **w***_t_* to **w**_0_ in the joint optimization problem via regularization. We use the following formulation, leaving out some constants for readability

where* l* is the hinge loss,* l*(*z, y*) = max{1 − *yz*, 0}.

It was shown in [15], that the dual formulation of the above corresponds to the standard SVM using a modified kernel function:

where *K_B_* denotes the base kernel that captures the interactions between examples from all tasks and

 (1)

Here,* t*(*i*) denotes the task of example *x_i_*. In the above formulation,  is composed of the general kernel *K_B_* and the kernel *δ_t_*_(_*_i_*_),_*_t_*_(_*_j_*_)_*K_B_*(*x_i_*, *x_j_*) that captures only intra-domain interactions. In [9], the latter kernel is referred to as* Dirac* kernel. A slightly more general formulation of  is the following, which allows to adjust the trade-off between the general kernel and the task-specific kernel:

where* β*_1_,* β*_2_ ≥ 0 and* β*_1_ +* β*_2_ = 1.

Clearly,  is a convex combination of base kernels and thus a valid kernel. MKL is a technique to learn the individual weights of a weighted linear combination of kernels. Thus, it seems natural to utilize MKL to learn an optimal weighting for .

### Multiple Kernel Learning

Lanckriet et al. considered conic combinations of kernel matrices for classification [16], leading to a convex quadratically constrained quadratic program. Later on, it was shown that the problem can be formulated as a semi-infinite linear program, allowing to use standard SVM solvers (e.g. SVMLight [17], LibSVM [18]) for solving the reoccurring sub-problems [13]. Only recently, methods were proposed to generalize MKL to an arbitrary *l_q_*-norm [19].

Learning with multiple kernels gives rise to *M* different feature mappings *ϕ_m_*: *X* → *H_m_*, *m* = 1,…, *M*, each leading to a kernel *K_m_* for a Hilbert space *H_m_*. In MKL, we consider linear mixtures of kernels , where *β_i_* ≥ 0. To avoid non-convexity, the original parameter vector  is substituted . For an in depth discussion of this, please consider [19].

We use the following formulation in the primal:

   (2)

where* l* is the hinge loss,* l*(*z, y*) = max{1 −* yz*, 0} and q denotes the norm used to penalize the weights *β*. To solve the above optimization problem, we follow ideas presented in [13],[19] to iteratively solve a convex optimization problem involving only the *β*’s and then to solve for **w** only. This method is known to converge fast even for a relatively large number of kernels [13].

## Multitask Multiple Kernel Learning

To be able to use MKL for Multitask Learning, we need to reformulate the Multitask Learning problem as a weighted linear combination of kernels. In the spirit of Equation 1, the basic idea of our decomposition is to define task-based block masks on the kernel matrix of *K_B_*. Given a list of tasks *T* = {*t*_1_,…,*t_T_*}, we define a kernel *K_S_* on a subset of tasks *S* ⊆ *T* as follows:

where* t*(*x*) denotes the task of example *x*. Here, each *S* corresponds to a* meta-task* as defined in the introduction. In the most general formulation, we define a collection *I* = {*S*_1_,…,*S_p_*} of an arbitrary number *p* of task sets *S_i_*, which defines the latent structure over tasks. This collection leads to the following linear combination of kernels, which is positive semi-definite:

Using , we can readily utilize existing MKL methods to learn the* β_i_.* This corresponds to identifying the groups of tasks *S_i_* for which sharing information leads to improved performance. After training using MKL, we have obtained a classifier* f_t_* for each task *t*:

where *N* is the total number of training examples of all tasks combined.

What remains to be discussed is how to define a collection *I* of candidate subsets *S_i_* (i.e. meta-tasks), which are subsequently to be weighted by MKL. We consider two scenarios, one where we assume to have access to a hierarchical structure relating the tasks at hand and one, where we assume no prior knowledge given about task relations. Generally, however, it is possible to utilize prior domain knowledge indicating how tasks are related to design an appropriate *I*.

### Powerset MT-MKL

With no prior information given, a natural choice is to take into account all possible subsets of tasks. Given a set tasks *T*, this corresponds to considering the power set *P* of *T* (excluding the empty set) *I_p_* = {*S*|*S* ∈ *P*(*T*) ∧ *S* ≠ Ø}.

Clearly, this gives us an exponential number (i.e. 2*^T^*) of task sets *S_i_* of which only a few will be relevant. To identify the relevant task sets, we propose to use an L1-regularized MKL approach (i.e. *q* = 1 in Equation 2) to yield a sparse solution. Most subset weights will be set to zero, yielding only a few relevant subsets with weights greater than zero. We expect that the examples in these subsets come from similar distributions and that it is therefore beneficial to consider interactions between them, when obtaining a multitask predictor.

While L1-regularization of MKL results in a sparse combination of kernels, it does not address the computational complexity of the optimization problem over this exponential search space. With the current implementation, the method is limited to approximately 10 tasks depending on the number of training examples and available resources. However, there are techniques to handle the case where the number of tasks may become prohibitive, for instance, as proposed in [20]. The idea is to iteratively generate new kernels based on the current solution (*β*, **w**). These methods are known to converge to the optimal solution, if one can identify appropriate kernels in a larger set. In the current case, this could be done by solving an integer linear program.

### Hierarchical MT-MKL

**Figure 1:**
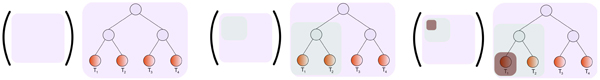
**Example of a hierarchical decomposition.** According to a simple binary tree, it is shown how each node defines a subset of tasks (a block in the corresponding kernel matrix on the left). Here, the decomposition is shown for only one path: The subset defined by the root node contains all tasks, the subset defined by the left inner node contains *t*_1_ and *t*_2_ and the subset defined by the leftmost leaf only contains *t*_1_. Accordingly, each of the remaining nodes defines a subset S*_i_* that contains all descendant tasks.

In the second scenario, we assume that we are given a tree structure *G* that relates our tasks at hand (see Figure 1). In this context, a task *t_i_* corresponds to a leaf in *G*. Assuming hierarchical relations between tasks is particularly relevant to Computational Biology where often different tasks correspond to different organisms. In this context, we expect that the longer the common evolutionary history between two organisms, the more beneficial it is to share information between these organisms in a MTL setting. We can exploit the hierarchical structure *G* to determine which subsets might play a role for Multitask Learning. In other words, we use the hierarchy to restrict the space of task sets. Let *leaves*(*n*) = {*l*|*l* is descendant of *n*} be the set of leaves below the sub-tree rooted at node *n*. Then, we can give the following definition for the hierarchically decomposed kernel function

As an example, consider the kernel defined by a hierarchical decomposition according to Figure 1. Clearly, the number of* β_i_* corresponds to the number of nodes. For a perfect binary tree this leads to 2*m* − 1 nodes, where m is the number of leaves/tasks. We expect that learning the contributions of the individual levels of the taxonomy makes sense for cases, where the edge lengths of *G* are unequal.

### Relation to task similarity

The learned weights* β_i_* reflect the importance of the subset *S_i_*. Clearly, if two tasks *t_k_* and *t_l_* are often jointly present in subsets with high weights, we expect those tasks to be similar to each other. One can infer a measure of pairwise similarities between tasks γ*_k,l_* from the weights* β_i_* of the subsets *S_i_*. We define the collection of task sets containing task *t_k_* as . Using this definition, we can define the similarity γ*_k,l_* between two tasks by summing up the weights of the shared task sets *S_i_*

  (3)

This similarity measure can be used for downstream analyses, as it provides insight about the task relationships. A high γ*_k,l_* between tasks suggests a considerable resemblance between tasks and could help to generate domain knowledge (e.g., evidence that two cell-receptors bind to similar class of proteins, or the molecular mechanisms of the splicing machinery particularly similar).

## Results and discussion

We performed experiments in two settings. In the first setting, we considered a set of MHC-I (major histocompatibility complex) proteins. Here, we assume we are not given any prior information to relate them. In the second setting, we used splice site data from 15 organisms and assumed that the task relationship is given by a hierarchical structure according to their evolutionary history. The examples are string data over an alphabet {*A*,*T*,*G*,*C*} (DNA) in the splicing case and the alphabet of 20 amino acids in the MHC-I case. To incorporate string features, we used the Weighted Degree String Kernel [21], which amongst other kernels such as the Spectrum Kernel [22], has been successfully employed in problems from Computational Biology.

In addition to the two MKL-based methods, we evaluated the following baseline methods:

*• Union* - One global model is obtained on the union of examples from all tasks.

*• Plain* - For each task, a model is trained independently, not taking into account any out-of-domain information.

*• Vanilla MTL* - Our algorithms consists of two components - the MTL formulation and the adjustment of weights* β_i_* with MKL. In the vanilla approach, we fix all weights at* β_i_* = 1.

Experiments were performed by using cross-validation for model-selection on the training splits. We only tuned one hyper-parameter* C,* for which we considered values between 0. 01 and 1000 on a logarithmic scale in 8 steps. After having obtained an optimal regularization parameter, a classifier is retrained on all training splits and final performance is obtained on a dedicated test set, that was not involved in hyper-parameter selection.

### MHC-I binding prediction using Powerset MT-MKL

In this experiment, the task is to predict whether a peptide binds to a certain MHC molecule (binder) or not (non-binder). It has been previously shown that sharing information between related molecules (alleles) and thus casting the problem in a Multitask Learning scenario, can be beneficial [9]. In the MHC setting, different tasks correspond to different MHC proteins. The data consists of peptide sequences of length *l* = 9 for 7 tasks. In total, we are given 7367 examples (A_2403=254, A_6901=833, A_0201=3089, A_0202=1447, A_0203=1443, A_2402=197, A_2301=104). For cross-validation, the data was split randomly into 5 splits of the same size. Unlike the setting of splice site prediction, we do not have a hierarchical structure relating our tasks at hand. To demonstrate that meaningful groups of tasks can be identified by Powerset MT-MKL, we do not assume any prior knowledge of task relationships. Please note, however, that we do have access to the sequences of the MHC-I proteins. We use these sequences to evaluate the task similarities obtained by our approach.

**Figure 2:**
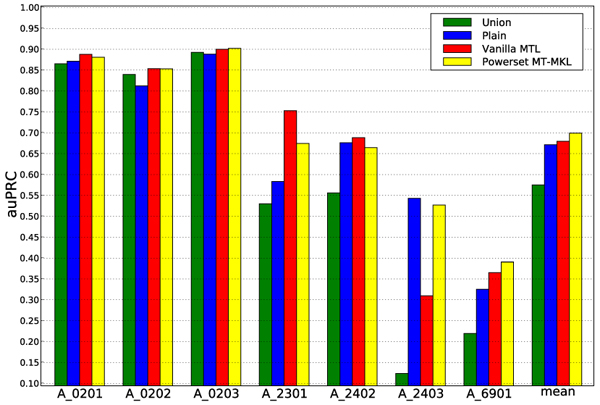
**Result for the MHC experiments.** Performance is shown for each of the 7 tasks. The performance averaged over all organisms is shown in the rightmost column mean.

We report the area under the precision recall curve (auPRC) for the individual tasks in Figure 2 and the summary of performances in Table 1.

**Table 1:** Results for the MHC experiment in auPRC for the model selection step and the final prediction on the test set. Reported is the average performance over all tasks.

auPRC	Plain	Union	Vanilla MTL	Powerset MT-MKL
cross-validation	0.668	0.637	0.676	0.692
test set	0.671	0.576	0.679	0.699

From Figure 2, we observe that the MKL-based approach outperforms the baseline methods. Furthermore, simply combining the data for different tasks to obtain a single model (*Union*) does not outperform the naïve method of obtaining an individual classifier for each task (*Plain*). This hints at rather large differences between the tasks. Learning the weights with MKL, improves performance compared to the* Vanilla MTL* approach, which already outperforms the other two baselines.

**Figure 3:**
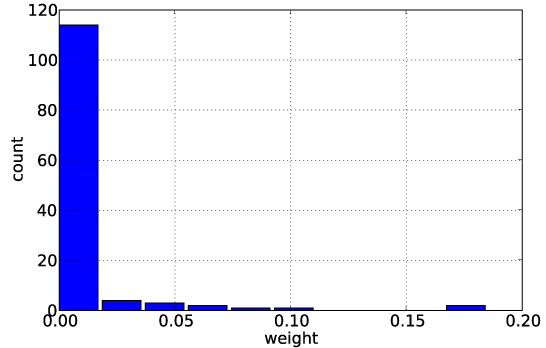
**Histogram of weights.** Shows the distribution of weights *β_i_* that are learned for the elements of the power set by MKL. As expected, most are (close to) 0.

**Figure 4:**
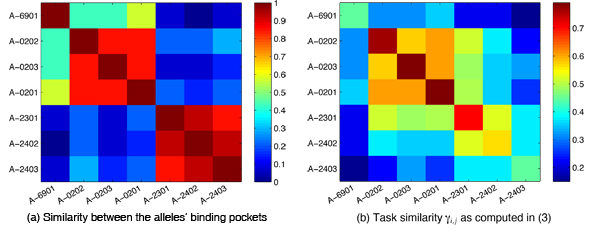
**Comparison between learned similarities and similarities based on the comparison of allele sequences.** The learned similarity of A-2301 with A-0203, A-0201 and A-0202 in (b) can be attributed to structural features that cannot be easily inferred from the allele sequence.

Figure 3 shows the distribution of weights obtained by the L1-regularized MKL approach. As expected, we observe that most task sets are assigned a weight of zero (or close to zero). Only a few get assigned a higher weight, so it is worthwhile to investigate the list of tasks that get assigned a weight *β_i_* > 0.05. From Table 2, we observe that the tasks* A_0201, A_0202, A_0203,* are often grouped in the same task set, which is in agreement with domain knowledge. Based on the assigned weights, we compute the task similarity as defined in Equation (3). For evaluation of the learned similarities, we compare them to the hamming distance (or similarity) between the amino acids in the binding pocket [23] of the MHC-I molecules (Figure 4). By visual inspection, we find a good agreement between the inferred task similarity and the molecule-based similarity.

**Table 2:** List of task sets and their respective weights *β_i_* that were assigned by 1-norm MKL.

Task Set					weight
A_0201,	A_0202,	A_0203,	A_6901		0.186
A_0201,	A_0202,	A_0203,	A_2301		0.178
A_0202,	A_0203,	A_2301,	A_2402,	A_2403	0.110
A_0201,	A_0203,	A_2301,	A_2402,	A_2403	0.091
A_0201,	A_0202,	A_2301,	A_2402,	A_6901	0.074
A_0201,	A_0202,	A_2301,	A_2402,	A_2403	0.066

Using MKL, we successfully identify groups of tasks among which information sharing is sensible, thus allowing for a smart combination of information from different tasks in the absence of prior knowledge.

The improvement in performance over the* Vanilla MTL* method is relatively small (a property most likely inherited from MKL). However, we are compensated for this by simultaneously obtaining a sensible task structure.

### Splice-site prediction using hierarchical MT-MKL

**Figure 5:**
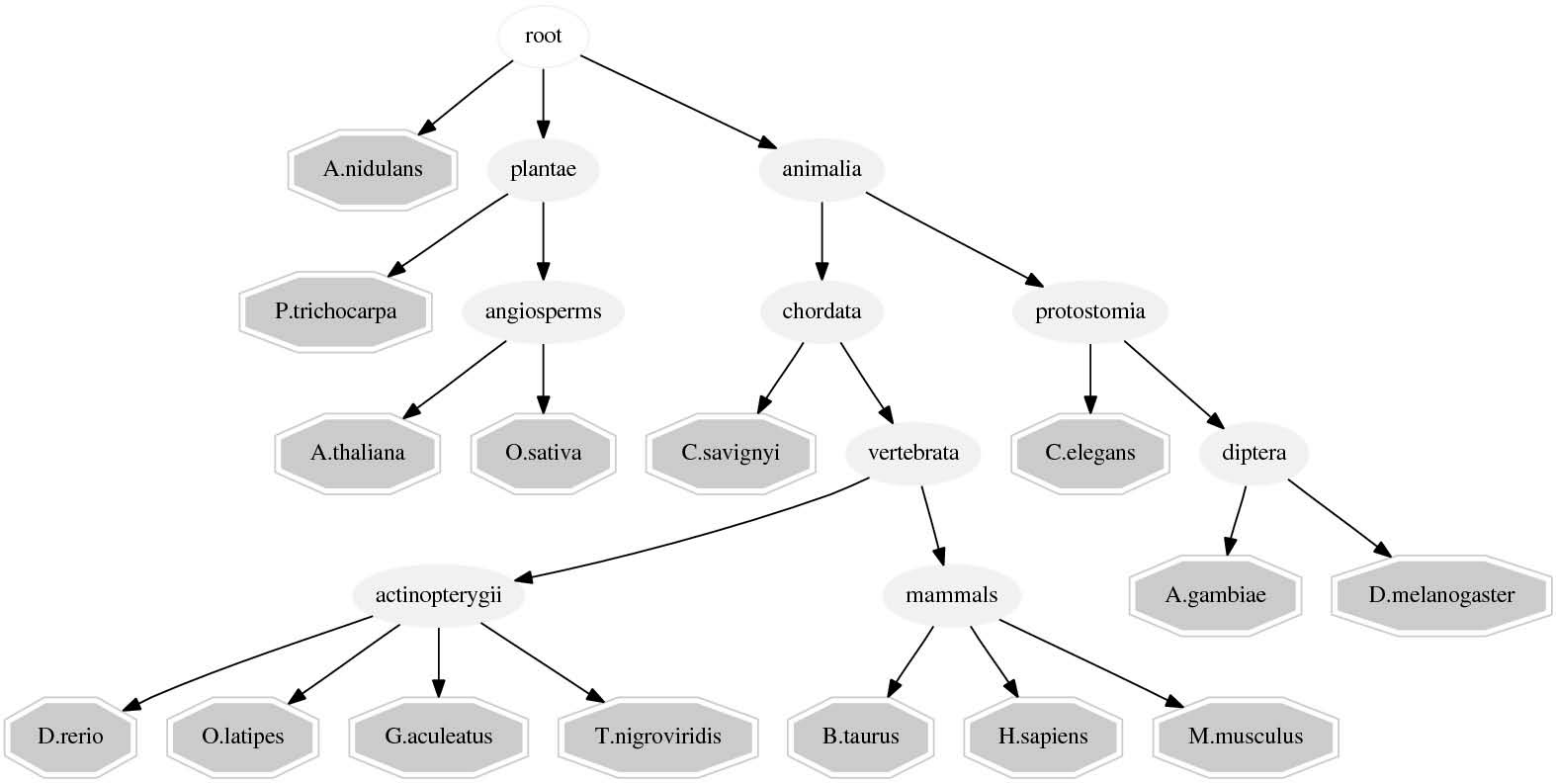
Hierarchical structure that defines the relationship between the organisms.

In this setting, we take into account a given hierarchy (see Figure 5) relating the 15 organisms in our data set. The data set consists of 6000 examples for 15 tasks, each at a positive to negative ratio of 1:100, similar to the one used in [3]. The data is split into 4 splits, three splits with 333 examples each and a large test split with 5000 examples. The dataset was created that way to establish a scenario where positive training examples are extremely rare.

We report the area under the precision recall curve (auPRC), which is well suited for unbalanced data sets. For the* Vanilla MTL* method, we use the given hierarchy *G* to define the initial task sets, but not further optimize their individual influence.

**Figure 6:**
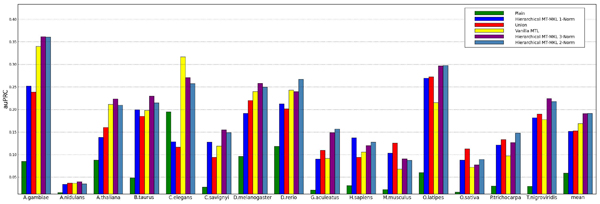
**Performance on test set for individual tasks.** The performance averaged over all organisms is shown in the rightmost column *mean*.

From Figure 6, we can make a few very interesting observations. First, in accordance with the results from the MHC-I experiment (see Table 3), the non-sparse Hierarchical MT-MKL methods outperform the baselines* Union* and *Plain*.

**Table 3:** Results for the splice site experiment in auPRC for the model selection step and the final prediction on the test set. Reported is the average performance over all tasks. This table shows only the performance for the best-performing variant of Hierarchical MT-MKL with norm *q* = 2.

auPRC	Plain	Union	Vanilla MTL	Hierarchical MT-MKL
cross-validation	0.043	0.092	0.087	0.100
test set	0.059	0.153	0.169	0.190

The second observation is that we get different results for different *q*-norms. In particular, we see a degraded performance for *q* = 1, which complies with our expectation that weights for this approach (assuming the hierarchy is correct) should not be sparse. For the *q*-norms that we considered, *q* = 2 performs best. Lastly, we can show that we are able to outperform the* Vanilla MTL* method (all* β_i_* = 1) by refining the task relations given by the structure *G* with MKL. Intuitively, using Hierarchical MT-MKL corresponds to estimating the edge lengths of *G*, whereas the other method is restricted to directly using the similarities encoded into the taxonomy.

## Conclusions

We have presented a principle way of formulating Multitask Learning as a Multiple Kernel Learning approach. Following the basic idea of task-set-wise decomposition of the kernel matrix, we present a hierarchical decomposition and a power set based approach.

These two methods allow us to elegantly identify or refine structure relating the tasks at hand in one global optimization problem. We expect our methods to work particularly well in cases, where edge weights differ within the hierarchical structure or where the task structure is unknown.

Our experiments illustrate that the MT-MKL approach on the power set of all tasks works well for the MHC binding problem: First it increases the accuracy of the predictors compared to the baseline methods and second, the inferred task similarity reflects the prior knowledge that is available for this problem. Also for the splice site prediction problem where the task hierarchy is given by the organisms’ phylogeny, our approach manages to achieve an improvement over standard approaches. Using MKL on top of regular Multitask Learning methods may uncover latent task structure and thereby provide insight into the problem domain, which might be relevant to downstream analyses. In conclusion, this work constitutes a valuable proof-of-concept outlining a principle way of using MKL to improve Multitask Learning.

## List of abbreviations used

MKL: Multiple Kernel Learning; MTL: Multi Task Learning; MHC: Major Histocompatibility Complex; SVM: Support Vector Machine; auPRC: area under the Precision Recall Curve.

## Competing interests

The authors declare that they have no competing interests.

## Authors’ contributions

Christian Widmer worked out the idea and implementation, performed the experiments and prepared part of the manuscript. Yasemin Altun was involved in the discussions, the development of methods on which this paper is based and the preparation of the manuscript. Nora C. Toussaint contributed to the discussions, provided the data for the MHC-I experiments and contributed to the preparation of the manuscript. Gunnar Rätsch came up with the original idea for the project and supervised the project at each step.
